# Nephrology Consultation for Severe SGLT2 Inhibitor-Induced Ketoacidosis in Type 2 Diabetes: Case Report

**DOI:** 10.3390/medicina55080462

**Published:** 2019-08-10

**Authors:** Felice Nappi, Antonietta La Verde, Giovanni Carfora, Carlo Garofalo, Michele Provenzano, Ferdinando Carlo Sasso, Luca De Nicola

**Affiliations:** 1 Nephrology Unit at S. Maria della Pietà Hospital, 80035 Nola (Naples), Italy; 2 Nephrology Unit at AORN Sant’Anna e San Sebastiano Hospital, 81100 Caserta, Italy; 3 Division of Nephrology, University of Campania “Luigi Vanvitelli”, 80137 Naples, Italy; 4 Division of Nephrology, University Magna Grecia, 88100 Catanzaro, Italy; 5 Division of Internal Medicine, University of Campania “Luigi Vanvitelli”, 80137 Naples, Italy

**Keywords:** diabetic nephropathy, SGLT2 inhibition, AKI, ketoacidosis, diabetes mellitus

## Abstract

Euglycemic diabetic ketoacidosis (euDKA) related to sodium-glucose cotransporter 2 inhibitor (SGLT2-I), despite being reported as consistent, though infrequent, adverse effect in all trials on SGLT2-I in type 2 diabetes mellitus (T2D), still remains poorly known in the real world. On the other hand, the use of this new class of antihyperglycemic agents is expected to increase based on the recent solid evidence of remarkable cardiorenal protection. Therefore, improving awareness on risk factors, diagnosis, and treatment of euDKA is essential to allow correct implementation of SGLT2-I in clinical practice. We here report a T2D patient admitted to the emergency department and then transferred to the nephrology-dialysis unit because of severe euglycemic diabetic ketoacidosis (euDKA) related to sodium-glucose cotransporter 2 inhibitor (SGLT2-I). In our patient, a concurrent acute kidney injury at presentation, initially attributed to excessive use of nonsteroid anti-inflammatory agents, and the absence of severe hyperglycemia led to delayed diagnosis and proper therapy. The detailed description of decision-making process for diagnosis and therapy, and the analysis of precipitating factors as well, discloses the helpful contribution of nephrologist to optimize prevention and management of euDKA.

## 1. Introduction

Diabetic ketoacidosis (DKA) is a major life-threatening event of type 1 diabetes mellitus that can, however, occur in type 2 (T2D) in the presence of relative insulin deficiency. A subtype of DKA, labeled “euglycemic” (euDKA), is characterized by plasma bicarbonate ≤10 mEq/L and glycemia ≤300 mg/dL [1]. In 2015, the Food and Drug Administration (FDA) issued a warning on the association between euDKA and inhibitors of sodium-glucose cotransporter 2 (SGLT2-I) [2]. The importance of the FDA black box has been recently supported by a large cohort study that confirmed that in T2D euDKA incidence is double in new SGLT2-I users versus dipeptidyl peptidase-4 inhibitors (4.9 events per 1000 person-years vs. 2.3 events per 1000 person-years), with two-fold risk after propensity-score matching [3]. Broadening of population under SGLT2-I is expected in the near future. These drugs are in fact now recommended as second-line therapy in T2D, especially in the population of diabetics with heart failure and/or chronic kidney disease (CKD) [4]. The interest of nephrology community on SGLT2-I and dependent euDKA will also grow based on the recent publication of results of CREDENCE (Canagliflozin and Renal Outcomes in Type 2 Diabetes and Nephropathy) in T2D patients with overt CKD [5]. This trial showed remarkable cardiorenal protection of canagliflozin; on the other hand, infrequent though higher incidence of DKA vs. placebo was noted (2.2 vs. 0.2/1000 pts-y). These data are in agreement with the findings of the recent systematic review of 34 case reports on euDKA from “real-world” [6], while do not support the conclusions of the meta-analysis of eight previous randomized controlled trials (RCTs) showing a neutral effect of SGLT2-I class versus other antihyperglycemic agents [7]. This discrepancy indicates a higher incidence of DKA in the real world as compared to the ideal setting of RCT unless renal dysfunction is present. Nevertheless, in clinical practice, this critical event is likely underdiagnosed, and the management still guided by weak evidence and consensus opinion [3,6,7,8,9,10,11].

## 2. Case Report

A 67-year-old woman with a 5-year history of T2D presented to the emergency department (ED) of a public hospital because of abdominal pain and impaired conscious level (Glasgow Coma Scale: 12), occurring after one week of fever, malaise, and dyspnea. Family reported that the patient was not a smoker nor was used to drink alcohol and had normal blood pressure. Clinical charts showed that she was free of cardiovascular disease and had albuminuria (800–1100 mg/g creatinine) with estimated glomerular filtration rate (eGFR) 75–85 mL/min/1.73 m^2^. In the last month, due to worsening glycemic control, the diabetologist prescribed a lower intake of calories and added empagliflozin 25 mg/day on top of usual treatment (metformin 1700 mg/day). The new therapy led to 14-kg body weight loss (from 67 to 53 kg). Family also mentioned bis-in-die therapy with nonsteroid anti-inflammatory agents (NSAID) for a joint pain throughout the two weeks before hospitalization. At presentation, BMI was 21.5 kg/m^2^. Vital signs were temperature 37.2 °C, pulse rate 110 beats/min, blood pressure (BP) 90/60 mmHg, respiratory rate 40 breaths/min, and urinary output 100 mL/h. Physical examination showed dehydration and no relevant findings at objective examination of lungs and abdomen. Table 1 reports main lab data during hospitalization.

In ED, diagnosis was “metabolic acidosis and hyperkalemia in diabetic patient with acute kidney injury (AKI) likely related to NSAIDs”, and treatment was intravenous (iv) bolus (140 mmol) of sodium bicarbonate 1.4% followed by continuous iv infusion of normal saline 0.9% and sodium bicarbonate 1.4% at the rate for either solution of 125 mL/h. In the subsequent 6 h, acidosis status slightly improved, thus leading to reduction of bicarbonate rate to 20 mL/h.

**Figure 1:**
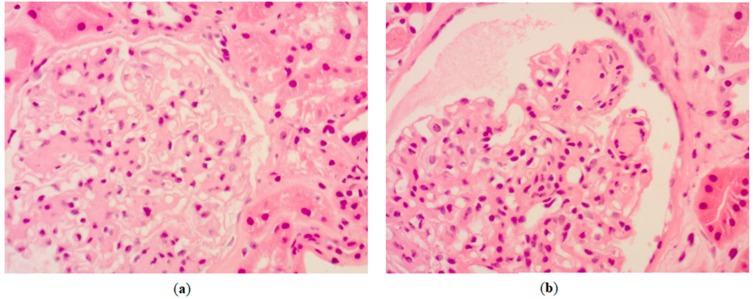
Hematoxylin-eosin staining of kidney biopsy specimen of the patient showing diffuse increase in mesangial matrix (**a**) and typical Kimmelstiel–Wilson nodules (**b**).

After 12 h of infusion, a renal consultant was called because of hypernatremia onset and persistent acidosis. Nephrologist asked for urinalysis, not included in the initial screening, that disclosed very high glucose and ketones levels. The consultant diagnosis was “AKI and DKA related to SGLT2-I”. Therefore, SGLT2-I therapy and bicarbonate infusion were discontinued, iv infusion of Lispro, a rapid-acting insulin analog (0.1 UI/kg/h) was started, and KCl 20 mmol/h was added to the normal saline infusion. In the same day, due to severity of acidosis and hypernatremia onset coupled with worsening of hemodynamics (BP 80/60 mmHg) and mental status, the patient was transferred to the nephrology department. She underwent 4-h treatment with bicarbonate hemodialysis for two consecutive days with ultrafiltration rate adjusted to allow volume expansion of 2.0 L/session. After the first session, urinary output increased up to about 500 mL/h due to repletion of extracellular volume, concomitant increase of BP (130/60 mmHg), and persistent glycosuria. Within few days, a significant improvement was observed in mental status, systemic hemodynamics, renal function, and hydroelectrolyte homeostasis. Insulin infusion continued for one week. Additional lab exams obtained in the nephrology ward demonstrated mycotic and bacterial infection of urinary tract associated with high procalcitonin (up to 7.7 ng/mL-normal <0.05 ng/mL) that was efficaciously treated by culture-guided therapy. Lab tests also showed high glycated hemoglobin (HbA1c 12%), very low fasting C-peptide (0.43 ng/mL), and absence of autoantibodies against insulinoma-associated protein 2 (IA-2 6.3 U/mL) and glutamic acid decarboxylase (GAD 4 U/mL) suggestive for latent autoimmune diabetes in adults (LADA). Following the complete resolution of the clinical picture, a kidney biopsy was performed at day 15 because of high albuminuria (942 mg/g) not readily attributable to diabetic nephropathy according to the relatively short duration of T2D and the absence of diabetic retinopathy. Histopathology, however, revealed overt diabetic nephropathy (Figure 1).

The patient was discharged after recovery of renal function, volemia, and metabolic control. Diagnosis was “euDKA related to SGLT2-I associated with AKI due to osmotic diuresis-induced volume depletion in patient with type 2 diabetic nephropathy”. The patient was switched on chronic insulin therapy and regular nephrology follow-up was indicated.

## 3. Discussion

In our patient, the whole picture was coherent with euDKA due to SGLT2-I (Table 2).

However, diagnosis was initially missed mainly due to the absence of urinalysis at admission. Indeed, the moderate hyperglycemia in euDKA has the deleterious effect of obscuring diagnosis as compared with “traditional DKA” characterized by severe hyperglycemia [2,10,11,12]. This case report differs from previous ones in four aspects that make it original: (1) The differential diagnosis between AKI-related metabolic acidosis and euDKA; in fact, an additional confounder in our patient was the concurrent AKI, although high anion gap acidosis purely related to kidney dysfunction rarely occurs when eGFR >20–25 mL/min/1.73 m^2^ and rarely associates with serum bicarbonate <14 mmol/L [13]. On the other hand, a potential role of metformin was easily excluded due to normality of lactate concentration [14]. (2) The extensive description not only of lab parameters but also of therapeutic approach. Importantly, misinterpretation of clinico-laboratory picture delayed proper treatment. The combined infusion of normal saline and bicarbonate over the first 12 h was in fact not efficacious on acidosis due to absence of the necessary continuous insulin infusion [8]. Besides being ineffective, the combined infusion in the presence of osmotic diuresis (persistent glycosuria) and poor intake of water (due to the confusional status) caused a net salt gain with development of hypernatremia that contributed to further worsening the clinical picture. Ketonemia in SGLT2-I-euDKA mainly is the combined effect of the imbalance of insulin-glucagon axis (↓insulin-↑glucagon) strictly related to glycosuria and increased tubular ketone reabsorption secondary to drug-induced volume depletion [1,12]. Of note, accelerated ketogenesis is expected in the case of severe restriction in carbohydrate availability or when insulin deficiency is more pronounced. Our patient had either precipitating factor because SGLT2-I was prescribed simultaneously with restriction of calories, and prescription was erroneously made in the presence of exhausted insulin reserve. (3) The availability of renal histopathologic study in this patient; interestingly, the severity of diabetic damage at kidney biopsy, which is compatible with prolonged poor metabolic control latter factor, supports the hypothesis of exhausted insulin reserve also testified by high HbA1c and very low fasting C-peptide. The “unexpected” histopathology findings highlight the major role of kidney biopsy for diagnosis and treatment in albuminuric T2D patients, even at older age [15]. (4) The prominent role of nephrologist in contributing to differential diagnosis and therapy of euDKA. euDKA due to SGLT2-I is still underappreciated in the real world of clinical practice; disseminating knowledge among all the specialists involved in the care of diabetic patients, including nephrologists, is essential to improve early recognition and appropriate treatment of this complication.

## 4. Conclusions

In conclusion, better knowledge of risk factors, diagnosis, and treatment of euDKA likely represent an essential step to translate the cardiorenal protective efficacy of SGLT2-I proven in RCTs [16] into full effectiveness in the real world of daily clinical practice. This case report suggests the need of more active involvement of nephrologist in the prevention and management of this critical event. Prior to hospitalization, kidney biopsy would have contributed to better risk stratification and possibly anticipated start of insulin therapy, and, more important, immediate intervention on volume depletion might have prevented AKI. At admission to ED, a prompt differential diagnosis of acidosis and immediate request of urinalysis would have allowed timely diagnosis and correct management of euDKA.

## Figures and Tables

**Table 1:** Main lab data at admission and during hospitalization.

Blood	(Normal Lab Values)	Admission	Hour-6	Hour-12	Day-7
Glucose, mg/dL	(70–100)	299	266	264	180
Na, mEq/L	(135–145)	139	152	157	136
K, mEq/L	(3.5–5.0)	5.9	3.1	2.9	3.5
Cl, mEq/L	(98–106)	108	116	117	98
Ca, mg/dL	(8.5–10.5)	9.31	-	8.8	-
Urea, mg/dL	(10–50)	42	-	65	22
Creatinine, mg/dL	(0.8–1.2)	1.70	-	1.80	0.8
_MDRD_eGFR, ml/min/1.73 m^2^	(>90)	31.9	-	29.8	76.0
AST U/L	(<35)	35	16	-	-
ALT U/L	(<35)	29	10	-	-
Amylase, U/L	(<100)	80	-	-	-
Hemoglobin, g/dL	(13–16)	10.5	-	10.0	9.8
Leucocytes, 10^3^ × mm^3^	(4.5–10)	17.1	NA	12.8	8.1
Neutrophils, %	(40–75)	91	NA	88	80
**Hemogasanalysis**					
pH	(7.36–7.44)	6.91	7.35	7.04	7.48
pCO2, mmHg	(35–45)	9	18	20	38
pO2, mmHg	(80–100)	138 *	123*	165 *	87
O2 saturation, %	(96–100)	99	95	98	98
HCO3, mmol/L	(21–28)	1.8	12.5	5.4	28.3
Lactate, mmol/L	(<4)	1.3	2.4	1.0	1.0
Anion Gap, mmol/L	(8–16)	31	24	35	10
**Urine**					
Ketones, mg/dL	(null)	-	-	80	0
Glucose, mg/dL	(null)	-	-	2000	2700

* Under O_2_ therapy.

**Table 2:** Sodium-glucose cotransporter 2 inhibitor (SGLT2-I)-induced euglycemic diabetic ketoacidosis (euDKA): clinical picture, diagnostic criteria, risk, and precipitating factors in the patient as compared with current knowledge [1,12].

SGLT2-I-induced euDKA	Patient
**Signs and Symptoms**	
Asthenia	Yes
Excessive thirst and urination	Yes
Vomiting and abdominal pain	Yes
Dehydration and hypotension	Yes
Changes in mental status	Yes
**Diagnostic Criteria**	
SGLT2-I utilization	Yes
Moderate hyperglycemia (less than 300 mg/dL)	Yes
Metabolic acidosis with high anion gap	Yes
Ketonemia and/or Ketonuria	Yes
**Risk factors**	
Excessive alcohol consumption	No
Type 1 DM or LADA	No
Down-titration or discontinuation of insulin	No
Low fasting C-peptide	Yes
Reduced intake of calories	Yes
Infections or intercurrent illness	Yes
Surgery	No
Acute cardiovascular events	No

LADA, latent autoimmune diabetes of adult.
